# Farming for Life Quality and Sustainability: A Literature Review of Green Care Research Trends in Europe

**DOI:** 10.3390/ijerph15061282

**Published:** 2018-06-17

**Authors:** Marina García-Llorente, Radha Rubio-Olivar, Inés Gutierrez-Briceño

**Affiliations:** 1 Department of Applied Research and Agricultural Extension, Madrid Institute for Rural, Agricultural and Food Research and Development (IMIDRA), Finca Experimental ‘‘El Encín’’Ctra N-II, Km 38, Madrid 28800, Spain; 2 Social-Ecological Systems Laboratory, Department of Ecology, Edificio de Biología, Universidad Autónoma de Madrid, C/Darwin 2, Madrid 28049, Spain; radha.rubio@gmail.com (R.R.O.); ines.gutierrezbriceno@gmail.com (I.G.B.)

**Keywords:** connective agriculture, nature-based rehabilitation, relational value, social inclusion, systematic literature review, therapeutic horticulture, vulnerable stakeholder

## Abstract

Green care is an innovative approach that combines simultaneously caring for people and caring for land through three elements that have not been previously connected: (1) multifunctional agriculture and recognition of the plurality of agricultural system values; (2) social services and health care; and (3) the possibility of strengthening the farming sector and local communities. The current research provides a comprehensive overview of green care in Europe as a scientific discipline through a literature review (*n* = 98 studies). According to our results, the Netherlands, the UK, Norway and Sweden followed by Italy have led the scientific studies published in English. Green care research comprises a wide range of perspectives and frameworks (social farming, care farming, nature-based solutions, etc.) with differences in their specificities. Green care studies have mainly focused on measuring the effectiveness of therapeutic interventions. Studies that evaluate its relevance in socio-economic and environmental terms are still limited. According to our results, the most common users studied were people suffering from psychological and mental ill health, while the most common activities were horticulture, animal husbandry and gardening. Finally, we discuss the potential of green care to reconnect people with nature and to diversify the farming sector providing new public services associated with the relational values society obtains from the contact with agricultural systems.

## 1. Introduction

Agriculture has been performed by our species for approximately 10,000 years [1], and practices have been altered according to human needs and preferences. The agricultural industrialization of the 20th century dramatically changed agricultural activities and relations between agriculture and our culture; for example, agriculture now focuses largely on the maximization of both production and profit [2]. This change has become even more severe over the last 50 years, during the green revolution, with the intensification of large-scale agricultural production and the abandonment of the countryside in traditionally agricultural rural areas [3,4].

The consequences of this transition not only has environmental impacts (i.e., loss of agricultural landscapes, water pollution, loss of genetic heritage related to local varieties and breeds), economic impacts (i.e., loss in profitability) and cultural impacts (i.e., loss of local knowledge and identity linked to agricultural management) but also affects our general nutrition, relationships, health and quality of life. The value and importance of the relation between humans and nature has been overlooked during recent decades. However, currently, it is known that contact with nature has a positive influence on quality of life in terms of both physical and psychological health [5,6,7]. Nevertheless, this disconnection (in terms of access and appreciation) with agroecosystems and the ecosystem services that agroecosystems provide is increasing in western and urbanized societies. It is argued by Pretty [8] that as urbanized societies we have become disconnected from the land that sustains us and we cultivate; thus, we are losing part of our culture and identity.

Human beings, as part of nature, have always coexisted with it; thus, the association between people and nature has always existed. This concept has been formalized in the academic world through the study of social-ecological systems [9]. Following the biophilia theory [10], this connection should be more important and integrated into our lives, but the ability to connect with and understand nature often depends on our experiences as children, and such experiences should be reinforced in our society [11]. In addition to the biophilia theory, Kaplan’s attention restoration theory [12] and Ulrich’s psycho-evolutionary theory [13] should be highlighted, as these theories defend and explain why and how our surrounding natural environment influences our lives and is important for us. In socio-cultural terms, the current individualist lifestyle in western societies has resulted in a disregard for social well-being, deriving in a disconnection from other people and lack of community [14]. According to Spain’s Millennium Ecosystem Assessment, in recent decades in urbanized societies’ good social relations have deteriorated, with a specific tendency toward the loss of social cohesion and an increase in individualistic, sedentary and isolated lifestyles [15]. These trends are reflected by various indicators such the number of people living alone and the amount of television consumption [3]. This situation affects the most vulnerable people in the system more dramatically, placing them at risk of social exclusion.

Green care is an approach that aims to combine, simultaneously, caring for people and caring for land. It promotes health and well-being for people at risk of social exclusion through the use of natural environments as the central element [14,16]. In green care, a series of activities are carried out in the context of agricultural and natural environments where activities and interactions with nature take place (i.e., activities performed on farms, orchards and gardens, forests, etc.) to produce physical, psychological, emotional, social, cognitive-educational, social and labor-integration benefits for people at risk of social exclusion [7,14,16,17,18]. At those interventions, diverse social groups could be involved, including elderly people, people with mental disabilities, people with various mental disorders or mental health problems (i.e., dementia, stress, anxiety, depression and schizophrenia), refugees, teenagers with problems, ex-prisoners, people with addiction or abuse problems, women suffering from male violence, people with various physical disorders (cancer, obesity, hearing impairment and other disabilities), migrants with difficulties, long-term unemployed people, persons belonging to ethnic minorities, etc.

Green care is an inclusive and umbrella term that includes a broad variety of interventions such as nature-based rehabilitation, care farming, social farming, therapeutic horticulture, animal-assisted intervention, etc. While these concepts are sometimes used as synonyms, all of them are sustained by different backgrounds and theories and have different representations in each country. In this study we will refer to the term green care in order to cover a broad area of research. Over recent decades, in many European countries, the use of agriculture as a tool of public health and social integration has been developed in different forms. Many projects and initiatives have arisen, with the existence of more than 170 care farms in the UK as of 2011 [19], nearly 600 care farms in the Netherlands as of 2005 [20], and nearly 700 social farms in Italy [17]. In this way, in many European countries, green care is a practice with a long history; however, numerous research projects and studies have been developed to formalize the concept only in the last decade. In fact, in 2007, a cost action called “COST Action 866 Green Care in Agriculture” was created as one of the first attempts to increase scientific knowledge of green care, as one of the main limitations of green care has been the lack of evidence about the effectiveness of its various practices [16].

Since the end of the 20th century and the beginning of the 21st century, there has been an increase in the number of scientific studies focused on green care throughout Europe. Therefore, the current paper uses Europe as a case study with the intention of better understanding the main research trends and pathways that have been taken in terms of green care development to obtain a comprehensive understanding of the progress and dimensions of this new discipline in Europe. The proposed specific objectives of this systematic review have focused on analyzing: (1) which countries have published more, within which approach and which research areas have been emphasized by studies related to green care; (2) the temporal evolution of these studies and the research objectives investigated; (3) the targeted populations of green care studies as well as the activities carried out with each population; and (4) the methods used for assessing green care interventions. Finally, we discuss how our analysis can contribute to future research and green care practices.

## 2. Materials and Methods

### 2.1. Search Procedure

The methodology of this study consists of a systematic review of the existing scientific literature on green care in Europe. Specifically, we gathered and selected all studies published in peer-reviewed journals via the search engine Web of Science. To encompass the spectrum of terminology used to refer to green care, we considered this term as well as all related terms that have been used. The complete list of English keywords included “care farm”, “ecotherapy”, “farm animal-assisted”, “gardening-based intervention”, “green care”, “horticultural therapy”, “nature-based rehabilitation”, “nature-assisted therapy”, “social farm”, “therapeutic garden”, “therapeutic horticulture”, “working in nature”. 

The search was restricted according to the following criteria: (1) all studies published until 2017 were included to avoid incomplete years (i.e., 2018); (2) original articles were from scientific journals to avoid double counting (and excluded short communications, letters to the editor or editorials, communications in congresses and reviews); (3) scientific articles were restricted to those published in English; and (4) scientific articles were published in European countries.

**Figure 1:**
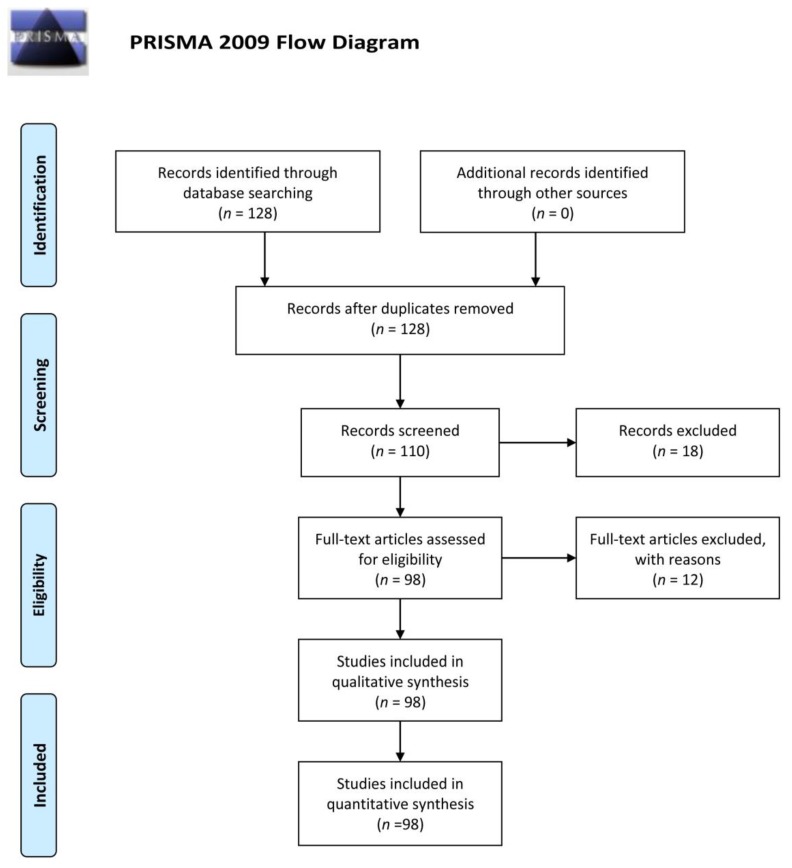
Flow diagram with the different phases of a systematic review (adapted from PRISMA, [21]).

Initially, 128 scientific articles were gathered in the search. Following the application of the above selection criteria and an inspection of the abstracts, 98 valid articles were selected (Supplementary material, Table S1). The remaining articles were excluded from the study because they did not meet any of the above criteria or because a read through of the publication indicated that they did not correspond to the topic in question (Figure 1).

### 2.2. Database Generation and Analysis

We extracted the following information from these publications: (1) publication identification (title, authors, year and journal); (2) discipline (level of disciplinary integration, i.e., uni-disciplinary or interdisciplinary, discipline area and research labels); (3) study characteristics (country studied, study type—theoretical or empirical); (4) study approach (following the terminology used in the study) and purpose; (5) target population; (6) type of activities conducted; and (7) methodological approach used to assess the intervention (when an intervention was implemented) (Table 1).

Regarding the purpose of publications, the published studies were classified into three main categories: (1) therapeutic assessments, including all the studies from the health sector that analyzed the effectiveness of different interventions; (2) concept, development and relevance of green care, including all the studies that practically or theoretically addressed the emergence of this new approach or aimed to define concepts, hypothesize potential benefits, or consider the impacts of its implementation; and (3) publications where the professionals were the cornerstone of the article and defined their preferences, views, needs to provide this health and social service as well as their networks (i.e., how are they organized).

First, we explored the current state of knowledge of green care through a general descriptive analysis of all included studies. To do so, we analyzed the countries that have published more studies, the theoretical framework used (care farming, nature-based rehabilitation, etc.), the field-specific disciplines related to the subject, the temporal evolution of the studies that included green care as their main research goal, the activities conducted, the main stakeholders and the methods used. Then, chi-square tests were performed to detect significant associations between specific variables. Specifically, chi-square tests were used to assess the relationship between countries and theoretical frameworks used, countries and discipline areas, countries and user types, countries and activities conducted, and finally, between activities and user types.

## 3. Results

### 3.1. Overview of the Scientific Studies on Green Care Carried out in Europe

**Figure 2:**
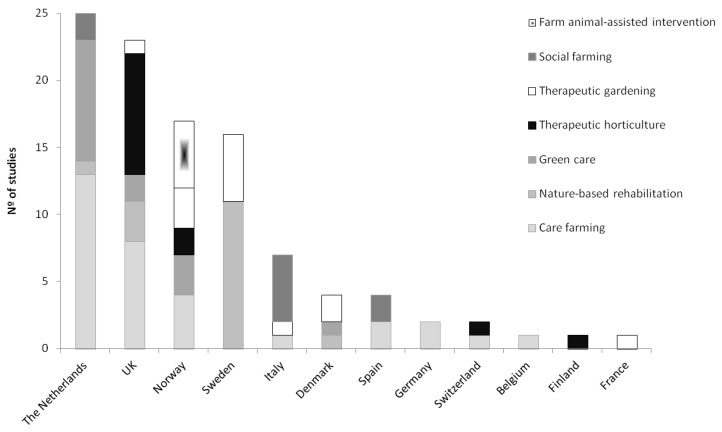
Number of publications per country, including the approach used.

A comparison of the studies published in different European countries showed that four countries led the scientific research on green care: the Netherlands (24%), the UK (22%), Norway (17%) and Sweden (16%). These top four countries were followed by Italy, which accounted for 7% of the publications, and other countries, such as Denmark, Spain, Germany, Switzerland, Belgium, Finland and France, which had low representation (approximately 1–4% each) (see Figure 2). The differences in the percentages of studies published in different countries may be due to the language restrictions used during the search process, as we analyzed only papers published in English.

Green care research comprises a wide range of perspectives and frameworks with differences in their specificities. In this regard, we identified seven different terminologies associated with those frameworks: care farming (used at 31% of the publications), nature-based rehabilitation (which includes forest interventions and ecotherapy, used at 16% of the publications), green care (15%), therapeutic horticulture (13%), therapeutic gardening (11%), social farming (8%) and farm animal-assisted interventions (5%). We detected significant differences performing chi-squared contingency-table test showing that some countries follow specific approaches. In this regard, the Netherlands used green care concept in its broadest sense more than other terms in their research studies (χ^2^ = 27.46; *p* < 0.05). In the UK most of the studies came from the therapeutic horticulture approach (χ^2^ = 21.64; *p* < 0.05). In Norway we found a significantly higher number of studies using the farm animal-assisted intervention approach (χ^2^ = 30.06; *p* < 0.05). Publications conducted in Sweden used the term nature-based rehabilitation significantly more than other terms (χ^2^ = 52.87; *p* < 0.05). Finally, studies from Italy used mainly the term social farming (χ^2^ = 35.46; *p* < 0.05) (Figure 2).

Most of the articles (63% of the studies analyzed) were interdisciplinary in nature, which allowed for a holistic approach to assessing the field of green care. Concerning the disciplines that assessed the subject of green care, health sciences and environmental sciences were the dominant areas (45% each of them), followed by social sciences (10%). In Europe, green care has been frequently framed in the field of health sciences (including areas such as rehabilitation, geriatrics and gerontology, occupational health, public health, psychiatry, dietetic and nutrition and oncology) and has included research on the therapeutic effects of green care and its impact on indicators of health and well-being. Such research includes publications on the impacts of therapeutic landscapes for older people [22], horticulture for clinical depression [23], and farm animal-assisted interventions for people with clinical depression [24]. From the environmental perspective (including researchers from the fields of vegetal science, agriculture, ecology and forest science), examples of published studies have focused on the values of landscapes and their management [25] or on the conceptualization of terms and the capacity of green care farms to promote social-ecological sustainability and ecosystem services [26]. A lower number of authors came from social sciences backgrounds emphasizing socioeconomic aspects; such as analyzing the economic impacts of green care, including indicators of expenditure and employment [27]; or investigating the evolution of rural social cooperatives engaged in green care farm practices [28]. When we performed the chi-squared contingency table test we detected significant differences, showing that the Netherlands and the UK were specialized in specific research areas. Such specialization was specifically seen in the Netherlands, where there was a predominance of studies coming from the environmental sciences (χ^2^ = 9.21; *p* < 0.05). In the UK most of the studies came from the health sector (χ^2^ = 11.88; *p* < 0.05), which is consistent with the therapeutic horticulture approach used with clear health goals defined (Figure 2).

### 3.2. Temporal Evolution of Green Care Studies and Their Research Objectives

**Figure 3:**
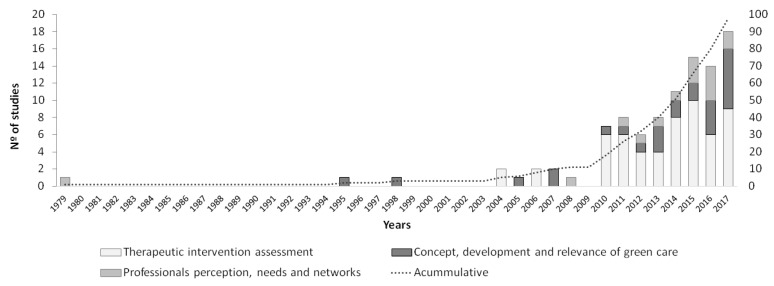
Temporal trends in published research by the study purpose.

The first study was published in the UK in 1979, and it focused on the requirements of horticultural training programs for people with mental health disabilities [29]. During the 1990s, two studies were published in relation to the concept, development and relevance of green care. These two theoretical studies were conducted in the health sector and explored the role of horticultural therapy [30] and gardens [31] in supporting people with disabilities, and they emphasized the elderly population. These types of studies had the purpose of providing confidence to caregivers regarding the use of green tools in human well-being interventions. Since 2004, a progressive increase in the number of studies has been observed, and this increase has been exponential since 2010 (Figure 3). In 2004, a network was created to promote knowledge sharing in European countries; it was the community of practice (Cop) “farming for health”. Later, in 2007, a project called “COST Action 866 Green Care in Agriculture” was launched, and it aimed to further investigate the concept of green care and its development in different European countries. The COST Action 866 Green Care Initiative was born in 2007 as a network in which researchers, engineers and scientists cooperated and whose main objective was to increase knowledge within the framework of green care. This project involved researchers from 22 countries, and it aimed to promote scientific knowledge in relation to green care, develop and deepen the concept, and highlight the potential of this new discipline in different European countries [16]). Thus, COST 866 was one of the first initiatives to formalize green care as a scientific discipline. Subsequently, at the scientific level, the European SoFar (Social Farming in Multifunctional Farms) project was financed by the Sixth Framework Programme during the 2006–2009 period. More recently, the SoFab Project (Social Farming across Borders) has been approved and implemented (2014–2017) in Ireland and Northern Ireland through INTERREG IVA Cross-border Programme funding. All these academic initiatives may explain the increase in the number of published studies.

Considering the general purposes of these publications, articles assessing health interventions have a long tradition, while studies exploring the concept, development and implementation of this discipline have been present but to a much lesser extent. During recent years, articles from the perspective of green care providers and how they are organized have become more visible (Figure 3). In our sample, we found that 58% of the studies were assessments on therapeutic intervention. Specifically, these studies from the health sector analyze the effectiveness of different treatments with different user types. Währborg et al. conducted a study comparing the effects of therapeutic gardening with the effects of conventional therapy on the rehabilitation of people suffering from depression or stress [32]. The results obtained after therapy concluded that people who had been treated in nature required less medical help than the other group. The study carried out by [33] aimed to evaluate whether the results of therapy that used activities in boreal forests could be utilized for the rehabilitation of patients suffering from exhaustion disorder. One of the results obtained suggested that the effect of this therapy is transitory, indicating that activities in nature should not be temporary in our lives; rather, these activities should be incorporated into our daily lives. The influence of contact with nature on children with attention deficit hyperactivity disorder was examined by [34]. The way in which women with stress-related illnesses experienced rehabilitation in a therapeutic garden was described by [35].

Then, 27% of the studies emphasized the concept, development and relevance of green care and included practical or theoretical publications that addressed the emergence of this novel approach; these studies aimed to identify the concepts and potential benefits, implementation possibilities and legislative frames that supported its implementation. These aspects differed by country, and many of these studies analyzed the evolution of green care in different countries that had their own particularities and trends, as seen by the evolution in the Netherlands [36,37], Flanders [36], Italy [28], and Switzerland [38]. Finally, in 15% of the publications, professionals were the cornerstone of the research, and they defined their preferences, views, need to provide this social service and health care, as well as their networks and organizational strategies and the benefits that they could obtain by including green care (mainly care and social farms) in their enterprises [39,40,41].

### 3.3. Target Population and Greem Care Activities

**Figure 4:**
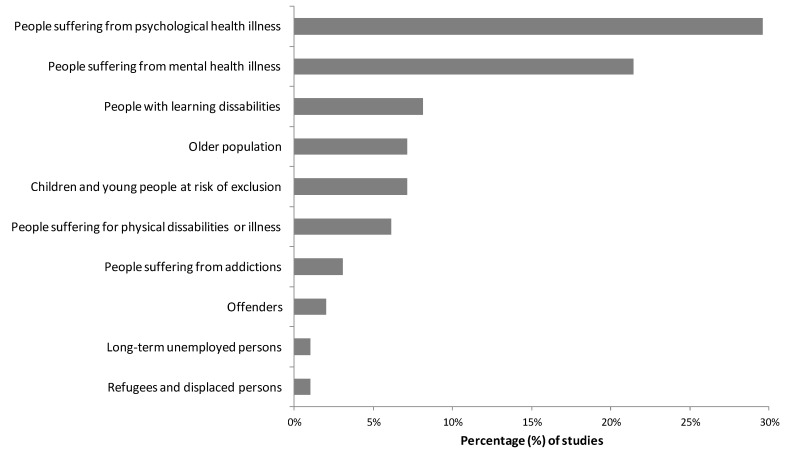
Type of users involved in green care programs.

Green care research covers a wide range of users who benefit from the interventions in which they participate. Following our sample, 10 categories of users have been identified, and two of these categories stand out (Figure 4): people suffering from psychological health illnesses such as depression, burnout and/or stress (e.g., [35,42]; in 30% of the studies), and people suffering from mental health illnesses, such as cases of dementia, schizophrenia, personality and behavioral disorders and other mental health problems (e.g., [43,44]; in 21% of the studies). Other publications focused on children and young people at risk of exclusion (e.g., those with behavioral problems or with dysfunctional family backgrounds; such as [11]; in 8% of the studies), on people with learning disabilities (e.g., [45]; in 7% of the studies), on elderly populations (e.g., [22]; in 7% of the studies), and on people suffering from physical disabilities or physical health illnesses (e.g., people with chronic muscle pain, coronary and pulmonary diseases or cancer; [46]; in 6% of the studies). Finally, a more limited number of studies focused on people suffering from addictions (4%), offenders (e.g., [47]; in 3% of the studies), people experiencing long-term unemployment (e.g., [48]; in 1% of the studies), and refugees and displaced people (e.g., [49]; in 1% of the studies).

Most of the studies were concentrated on a particular type of user (in 90% of the studies). We found a higher number of studies on people suffering from mental health illnesses in the Netherlands than in other countries (χ^2^ = 4.71; *p* < 0.05). We found a significantly higher number of studies focused on people suffering from psychological health illnesses in Sweden than in other countries (χ^2^ = 23.67; *p* < 0.001). Finally, we found a significantly higher number of studies focused on people with learning disabilities in the UK than in other countries (χ^2^ = 7.74; *p* < 0.05).

**Figure 5:**
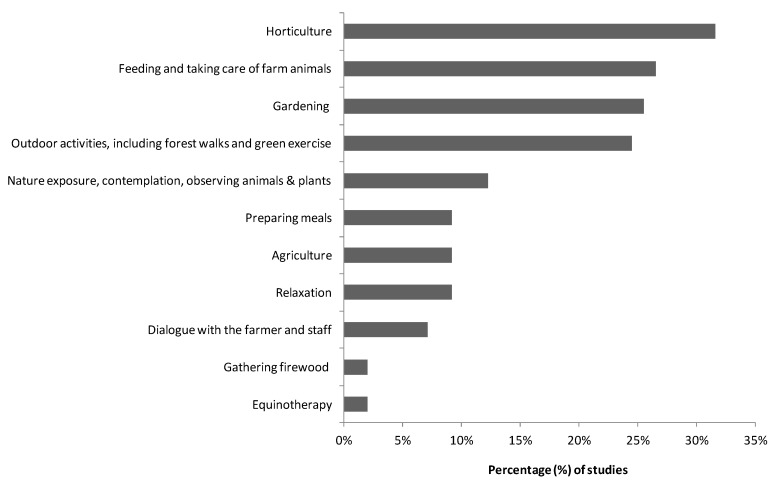
Green care activities carried out during interventions.

A wide variety of activities and tasks have been analyzed in the literature review conducted. Horticulture stands out as the most widely performed activity (32%), followed by animal husbandry by feeding and taking care of farm animals and working in stables (27%), gardening (26%), and outdoor activities, such as forest walks and other physical activities in green spaces (24%; Figure 5). Other types of activities that were carried out included being in contact with nature (e.g., passive exposure to vegetated environments) and contemplation (12%); food processing, cooking and preparing meals from farm products for sale (9%); agriculture production, including viticulture and olive orchards (9%); relaxation (6%); conversation with the farmers, other staff and the farm community (7%); firewood collection (2%) and equine-assisted therapy (2%). There were also mentions of training and educational activities through combined workshops (e.g., textile, carpentry, ceramics and art) focused on agricultural education and user training for labor market integration.

In 60% of the publications analyzed, a unique principal activity was studied, with 27% of the studies having two or three activities and 13% of the publications describing more than four activities. The typology of activities also differed from country to country in some cases, and we found a higher number of studies on engaging in outdoor activities (χ^2^ = 6.73; *p* < 0.05), relaxation activities in nature (χ^2^ = 18.38; *p* < 0.001) and gardening (χ^2^ = 6.03; *p* < 0.001) in Sweden than in other countries. Gardening was significantly more studied in the UK than in other countries (χ^2^ = 5.11; *p* < 0.05). In addition, Norway and the Netherlands produced more studies related to animal-assisted interventions, including activities such as animal husbandry (χ^2^ = 7.36 and χ^2^ = 5.25, respectively; *p* < 0.05). Finally, we tested associations between activities and user types. Following chi-square tests, we found that research publications studied the impact of relaxation activities on people suffering from psychological health illness (χ^2^ = 7.00; *p* < 0.05).

### 3.4. Methodological Tools for Assessing Green Care Interventions

**Figure 6:**
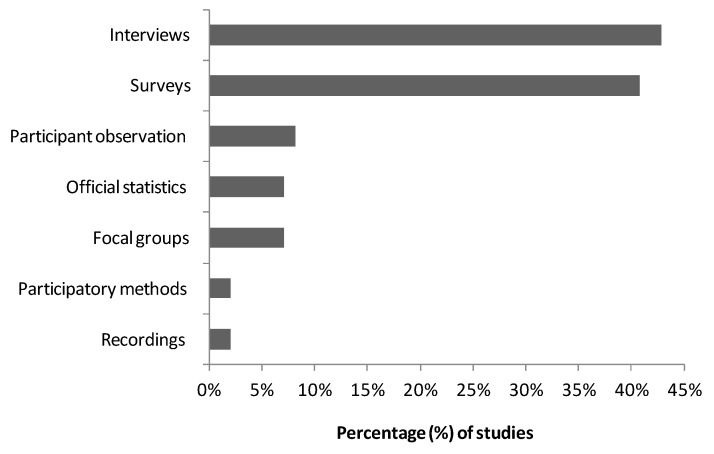
Methodological tools to assess intervention effectiveness.

The most common methods used to evaluate green care interventions were interviews (43%) and surveys (41%; Figure 6). Interviews involved semi-structured guides and open-ended questions to explore users’ experiences with green care practices. This was the case in the work conducted by [50], who analyzed forest-based rehabilitation through semi-structured interviews and analyzed the results from the perspective of the grounded theory. Interviews were carried out by [51] with care farmer professionals to explore the characteristics of diverse types of care farms in the Netherlands. Interviews were conducted by [52] with therapeutic garden users who had stress-related disorders to explore how they experienced the rehabilitation process.

Other studies have used quantitative data collected from questionnaires using experimental or quasi-experimental designs at clinical assesments. How a woodland program improved the psychological well-being of members of deprived urban communities was assessed by [53] using the perceived stress scale. Horticultural therapy as a physical health, mental health and social interaction with patients with chronic musculoskeletal pain was used by [54]. They used an experimental design and assessed indicators measured by the West Haven-Yale multidimensional pain inventory or the hospital anxiety and depression scale. A lower number of other studies gathered information from participant observations (8%), official statistics (7%), focal groups (7%), participatory methods (2%) and recordings (2%).

## 4. Discussion

### 4.1. Overview of Green Care Discipline across Europe

This study builds on previous literature reviews of green care interventions. A literature review was completed by [55] (*n* = 38 studies) on nature-assisted therapy that used controlled and observational studies to evaluate the scientific evidence, while five other publications were dedicated to specific user groups. The impacts on military veterans of sport and physical activity, including nature-based physical activities, were analyzed by [56] , (*n* = 11 studies). In the same way, [57] focused their literature review (*n* = 20 studies) on military veterans suffering traumatic experiences after active service and their participation in nature-assisted therapies. The evidence on the effectiveness of farm-based interventions for people with mental health disorders was reviewed by [58] (*n* = 11 studies). The benefits of gardening-based mental health interventions was evaluated by [59] (*n* = 10). Regarding dementia care, Whear et al. used qualitative and qualitative studies to examine the impacts of gardens and outdoor spaces on people with dementia ([60]; *n* = 17 studies), while González et al. evaluated the benefits of sensory gardens and horticultural activities ([61]; *n* = 16). Those reviews aimed to evaluate evidence that supported the effectiveness of green care interventions to significantly improve public health, mainly the health of specific users. Finally, a descriptive review was conducted by [62] of research on care farms for adults with mental health problems in Norway.

This research provides the first attempt to complete a comprehensive review of green care as a scientific discipline and includes studies assessing not only the effectiveness of interventions from the perspective of health but also other key aspects that require scientific attention, such as the concept, development and relevance of green care, as well as publications where professionals’ preferences, views, needs and networks were explored. Here, we analyzed trends in green care research using 98 publications that were conducted in different European countries. Although this study covered all Europe, we specifically reviewed scientific articles published in English. This limit provided a systematic method of searching for publications and avoided duplication; simultaneously, there was a limitation imposed by not collecting works published in other languages. For instance, there is evidence concerning the situation of green care under the approach of social farming in Catalonia in studies written in Spanish [63] or Catalan [64]. Much research conducted in Italy, mainly within the framework of social farming, has been published in Italian [17]; thus, such research has been underrepresented in the current study. In addition, Pawelczyk et al. attributed the lack of knowledge and research in Poland to the lack of knowledge about the usefulness of farming activities as a tool for tackling socio-health problems [65].

In this study we decided to use the broadest framework (green care) in order to cover the larger number of studies developed in Europe. However, as presented in our findings and also pointed out by other authors, there is a diversity of terms associated with different interpretations of the synergy between being in contact with natural and agricultural landscapes and the promotion of health together with other quality of life dimensions (e.g., employment, good social relationships, equity, education) [14,48,66]. Here, we identified seven terms used in research publications, the most popular being care farming. Nevertheless there were differences among countries, for example the green care term was used more in the Netherlands research, therapeutic horticulture approach in UK, farm animal-assisted intervention in Norway, the concept of nature-based rehabilitation in Sweden, and studies from Italy mainly used the term social farming. Some of the differences between those approaches are in the level of care and therapy provided [16]. Those interventions done within structured rehabilitation or health programs with clearly defined patient-orientated goals are commonly defined with the terms therapy or care such as therapeutic horticulture, therapeutic gardening or care farming [67]. In care farming and social farming the objectives are more related to conducting meaningful occupational activities and achieving employment goals at real production and commercial farms [40] and especially within social farming the therapeutic intent is not so explicit, with the aim being to promote innovation and collaboration pathways between sectors in local communities following social and employment inclusion and integration principles [68]. Other studies differ by the key element or tool used during the intervention, such as being in contact with nature at outdoor surroundings (at nature-based rehabilitation, [50]) or farm animal-assisted therapy being essential to the interactions established with animals (such as empathy, expression emotions or not being judged; [69]). In this way, green care is an umbrella term that represents a complex interaction between nature–people with different goals and specificities that determines the formalization of the approach. Green care is a dynamic concept, that has developed rapidly during the last 10 years and that will continue in progress as it represents a mirror of the different European countries and societies in terms of its culture, path dependence, needs and future expectations. This study reflects the green care research trends, giving the opportunity to offer an overview of the recent years and present time and to draw conclusions for the future. As presented by Di Iacovo et al., in Europe there are two models derived from two welfare systems: the northern European specialized model and the Mediterranean communitarian one. While in the first (followed by countries such as Sweden and the Netherlands), green care farms provide a health service (delivered by specialized facilities and skills) in private farms they receive direct payments (from the state or from the market being directly paid by users) for those services [70]. In the Mediterranean model (e.g., Italy or Spain) usually farmers do not receive a direct payment but receive other benefits more related with enhancing their reputation and expanding their networks. In this model, the goals pursued are more linked to social inclusion and justice than therapy. This situation may also explain the larger number of studies found in northern countries compared with those in the Mediterranean area; the number of studies being higher when the green care activities are more explicitly defined and where it is essential to measure the therapeutic effectiveness of the interventions conducted. In fact, there are small cooperatives or enterprises operating at the agrifood sector sustained by social economy and following agroecological principles (e.g., community supported agriculture) which are closely connected with social farming (e.g., justice, inclusion, solidarity, promotion of rural economies) but this is not explicitly stated, and it would be interesting to study the association between both approaches.

### 4.2. Target Population and Green Care Activities

It has increasingly been seen that green care responds to the needs of diverse groups, such as the training and working skills required by people who have experienced long-term unemployment or low employability, and the social integration of marginalized communities or spaces for community dialogue and interaction [71]. It improves not only their health but also their physical, psychological and emotional well-being (e.g., [55]). Green care provides opportunities to allow people to actively participate in society and agricultural landscape conservation. Green care has the potential to stress the relationships established between people and nature, uncovering the relational values obtained from agricultural landscapes. In an increasingly urban society, spending time in more natural, greener and more rural environments can help to meet new food, labor and social needs [72]. It has been proposed that to go beyond the classical duality to sustain landscape conservation based on intrinsic vs. instrumental values, policies should take into consideration relational values derived from the relationships establish between people and nature (e.g., cultural identity, stewardship principles), including relationships that are between people but involve natural surroundings (e.g., social cohesion) [73]. Active exposure to nature can promote a healthier lifestyle in the long term, which can help people cope with the effects of rapid lifestyles experienced in cities (e.g., stress, depression, fatigue) and address problems (quality food or lack of physical activity) facing people with increasingly sedentary futures [74].

Regarding green care activities, according to our findings, the most researched activities are horticulture, feeding and taking care of farm animals, gardening activities and outdoor activities, such as forest walks and green exercise. We found some significant associations between users and activities. In this regard, [75] analyzed different green care farming activities in terms of their suitability for different type of users taking into account aspects such as previous knowledge needed, need of support, risk due to the use of tools, etc. It would be a step forward to carry out further research to connect practices and specific well-being objectives to reach different users.

## 5. Conclusions

Some of the difficulties that a new science, movement and practice such as green care can face include gaining scientific, political and social credibility. Despite the advances in research publications, the potential of green care is still poorly understood [19,38]. As shown, in the last decade, researchers have started to study the effectiveness of green care compared to other therapeutic processes. However, since green care (mainly its orientation through social farming) contributes to rural revitalization—and the conservation of the agricultural landscape—it requires more scientific research that evaluates its relevance in socio-economic and environmental terms. It has been stressed that there is a need to recognize the complexity of views required to evaluate green care and to go beyond health indicators, since the mainstream measures of those indicators could mask and underestimate key components necessary to assess the development of green care practices (e.g., management procedures, networks of actors involved, certifications, consumer knowledge and acceptance of green care farms products, private or public policies to support them, etc.) [76]. Further research that proposes indicators and measures to analyses it as an innovative practice to diversify the farming sector, conserve agricultural landscapes and improve human well-being is required to ensure its establishment. In this regard, green care farming can be a major source of income for farmers [19,20] and a way to increase their visibility and reputation [26], which can stimulate the economy of the sector. It is necessary to determine which strategies farmers use, whether they are sufficiently innovative and whether they favor economic development [77]. It is also important to analyze the key factors that contribute to the success of green care projects by focusing on the point of view of producers and their willingness to innovate [40]. According to our findings, during recent years, the number of publications from the perspective of green care providers has been increasing (Figure 3). A shift in production models on farms can attract new types of workers by offering diversified activities through other approaches, skills, interests, benefits and resources that break with traditional farming and livestock activities. The diversification of agricultural activities can offer farm owners opportunities to provide new services. Green care, together with agro-tourism, has also been seen as motivation for women to diversify farming activities and promote female succession in farm properties in Norway, helping to counterbalance the masculinization of rural areas [78]. This can provide an incentive to significantly halt population declines in rural areas and could stimulate an increase in the number of women owners at the head of green care activities that occur on farms.

Green care activities can play a key role in enhancing life quality and sustainability in rural areas by providing economic and social benefits, as seen by recent cases of rural social cooperatives that have emerged in Italy [28]. Such cooperatives create a new relationship between urban and rural areas, as urban people are attracted to local markets in which they can find organic and ethical products with added social value. As was shown by [79], people were willing to support a green care initiative in the UK and were willing to contribute their money and voluntary time. Similarly, Carbone, A. et al. found that consumers’ buying groups in short food supply chains in Italy hold a strong concern for ethical issues when purchasing products and had an interest in supporting social farming products [80]. Unlike other economic sectors, agricultural activity can be understood as a transversal field with the capacity to influence a diversity of well-being components, not only in terms of production but also in terms of nutritional, educational, social and relational components as well as a new way of understanding the food system and our relationship with natural environments. This viewpoint aims to intensify social capital over intensive technological capital. From this perspective, farmers are essential actors since they can provide new services to society.

## Figures and Tables

**Table 1:** List of variables extracted from the database.

Variable Type	Variable	Description	Variable Coding
Publication identification	Title	Title of the publication	Open, text.
	Authors	Author (s) of the publication	
	Year	Year in which the research was published	Continuous (year).
	Journal	Name of the journal in which the article was published	Open, text.
Discipline	Interdisciplinary	Interdisciplinary team if at least two of the authors belonged to different research areas	Interdisciplinary; unidisciplinary.
	Discipline area	Based on the institution’s department and discipline where the first author works, we identified three main categories	Environmental Sciences; Science of the Health; Social Sciences.
	Research area (journal)	Based on Web of Science labels (one or more)	Categories.
Study characteristics	Study site	European country where the research was carried out	Dummy per each country.
	Type of study	Theoretical or empirical	Dummy for each category.
Study approach and purpose	Approach	Categories of existing theoretical frameworks following the terminology used in the study	Green care; nature-based rehabilitation; care farming; social farming; therapeutic horticulture; therapeutic gardening; farm animal-assisted intervention.
	Purpose	Categories of purposes pursued	Therapeutic intervention assessment; concept, development and relevance of social farming; professionals perception, needs and networks.
Users	Target population	Collective at risk of social exclusion on which the research is focus	Refugees and displaced persons; long-term unemployed persons; offenders; people suffering from addictions; people suffering for physical disabilities or illness; older population; people with learning disabilities; children and young people at risk of exclusion; people suffering from mental health illness; people suffering from psychological health illness.
Activities	Activity performed	Social agriculture activities carried out by participants benefiting from interventions	Outdoor activities (including forest walks and green exercise); agriculture (horticulture, viticulture and olive growing); gardening; therapeutic activities with animals; animal care; food processing and sale; nature exposure; relaxation; dialog with the farmer and staff.
Methodological approach	Assessment tools	Type of method used if there has been a follow up or assessment of participants of an green care intervention	Official statistics; surveys; interviews; focus groups; participant observation and participatory methods; clinical assessment; recordings.

## Supplementary Materials

The following are available online at http://www.mdpi.com/1660-4601/15/6/1282/s1, Table S1: Publications included in the systematic review.
