# Supplementary material for: Farming for Life Quality and Sustainability: A Literature Review of Green Care Research Trends in Europe
Source: Int J Environ Res Public Health. 2018 Jun 17;15(6):1282. doi: 10.3390/ijerph15061282 (PMC6025610; doi:10.3390/ijerph15061282)
Supplement: Supplementary file 1 [file ijerph-15-01282-s001.pdf]

Supplementary material

Table S1. Publications included in the systematic review.

Title	Authors	Publication year	Journal
Horticultural therapy- aspects of land use for the mentally handicapped system of planning for the requirements of the mentally-handicapped gardener	Spurgeon and Underhill	1979	International Journal of Rehabilitation Research
Horticultural therapy: Horticulture´s contribution to the quality of life of disable people	Stoneham et al.	1995	Acta Horticulturae
Therapeutics gardens	Ousset et al.	1998	Archives of Gerontology and Geriatrics
Plants in health care environments: Experiences of the nursing personnel in homes for people with dementia	Rappe and Lindén	2004	Acta Horticulturae
'Cultivating health': therapeutic landscapes and older people in northern England	Milligan et al.	2004	Social Science and Medicine
The paradox of the “green” prison: Sustaining the environment or sustaining the penal complex?	Jewkes and Moran	2005	Theorical Criminology
Day care for demented elderly in a dairy farm setting: Positive first impressions	Schols et al.	2006	Journal of the American Medical Directors Association
The association between indoor plants, stress productivity and sick leave in office workers	Bringslimark et al.	2006	Acta Horticulturae
Current status and potential of care farms in the Netherlands	Hassink et al.	2007	Wageningen Journal of Life Science
Values of rural landscapes in Europe: inspiration of or-product?	Pedroli et al.	2007	Wageningen Journal of Life Science
Attitudes to animal-assisted therapy with farm animals among health staff and farmer	Berget et al.	2008	Journal of Psychiatric and Mental Health Nursing
Day care at Green Care Farms: A novel way to simulate dietary intake of community–dwelling older people with dementia?	De Bruin et al.	2010	The Journal of Nutrition, Health, Aging
An exploratory study of the rehabilitation process of people with stress-related-disorders	Eriksson et al.	2010	Scandinavian Journal of Occupational Therapy
Therapeutic horticulture in clinical depression: a prospective study of active components	González et al.	2010	Journal of Advanced Nursing
Care farm in the Netherlands: Attractive empowerment oriented and strength based	Hassink et al.	2010	Health and Place Practices in the Community
Effects of an indoor foliage plants intervention on patient well-being during a residential rehabilitation program	Raanaas et al.	2010	Hort Science
The development of green care in western European countries	Haubenhofer et al.	2010	Explore-the Journal of Science and Healing
Gardening and belonging: reflections on how social and therapeutic horticulture may facilitate health, wellbeing and inclusion	Diamant and Waterhouse	2010	British Journal of Occupational Therapy
Social co-operatives and social farming in Italy	Fazzi	2011	Sociologia Ruralis
Impact of support centres for social farming on benefits from livestock in northern Europe	Schuessler et al.	2011	Outlook on Agriculture

Title	Authors	Publication year	Journal
A prospective study of existential issues in therapeutic horticulture for clinical depression	González et al.	2011	International Journal of Mental Health Nursing
Comparing day care at green care farms and at regular day care facilities with regard to their effects on functional performance of community-dwelling older people with dementia	De Bruin et al.	2011	Dementia
Experiences of women with stress-related ill health in a therapeutic gardening program	Eriksson et al.	2011	Canadian Journal of Occupational Therapy-revue Canadienne D
Animal-assisted therapy with farm animals for persons with psychiatric disorders	Berget and Braastad	2011	Annali dell´Istituto Superiore di Sanita
Farm animal-assisted intervention: relationship between work and contact with farm animals and change in depression, anxiety, and self-efficacy among persons with clinical depression	Pedersen et al.	2011	Issues in Mental Health Nursing
A comparison of children with ADHD in a natural and built-setting	Van den Berg and Van den Berg	2011	Child Care Health and Development
Care farms as a short-break service for children with Autism Spectrum Disorder	Van Zoneveld et al.	2012	Wageningen Journal of Life Science
Care farms in the Netherlands: An underexplored example of multifunctional agriculture-toward an empirically grounded, organization theory-based typology	Hassink et al.	2012	Rural Sociology
Important elements in farm animal-assisted interventions for persons with clinical depression: A qualitative interview study	Pedersen et al.	2012	Disability and Rehabilitation
The value of an allotment group for refugees	Bishop and Purcell	2012	British Journal of Occupational Therapy
Primary-care based participatory rehabilitation: User´s view of a horticultural and arts project	Barley et al.	2012	British Journal of General Practice
Horticultural therapy for patients with chronic musculoskeletal pain: Results of a pilot study	Verra et al.	2012	Alternative Therapies in Health and Medicine
The economic impact of agriculture in Dutch regions: An input-output model	Heringa et al.	2013	NJAS-Wageningen Journal of Life Science
Stress rehabilitation through garden therapy: The garden as a place in the recovery from stress	Adevi and Mårtensson	2013	Urban Forestry and Urban Greening
Multifunctional agriculture meets health Care: Applying the multi-level transition sciences perspective to care farming in the Netherlands	Hassink et al.	2013	Sociologia Ruralis
Multifunctionality and care farming: Contested discourses and practices in Flanders	De Krom and Dessein	2013	NJAS-Wageningen Journal of Life Science
Investigating the limits of multifunctional agriculture as the dominant frame for Green Care in agriculture in Flanders and the Netherlands	Dessein et al.	2013	Journal of Rural Studies
Mental health recovery on care farms and	Iancu et al.	2013	Disability and

Title	Authors	Publication year	Journal
day care centres: a qualitative comparative study of user´s perspectives			Rehabilitation
Does a structured gardening programme improve well-being in young onset dementia? A preliminary study	Hewitt et al.	2013	British Journal of Occupational Therapy
How effective is the Forestry Commission Scotland's woodland improvement programme 'Woods In and Around Towns' (WIAT)—at improving psychological well-being in deprived urban communities? A quasi-experimental study	Silveirinha de Oliveira et al.	2013	BMJ Open
The effects of social and therapeutic horticulture on aspects of social behaviour	Sempik et al.	2014	British Journal of Occupational Therapy
Outdoor environments in healthcare settings: A quality evaluation tool for use in designing healthcare gardens	Bengtsson and Grahn	2014	Urban Forestry andUrban Greening
Farming with care: the evolution of care farming in Netherlands	Hassink et al.	2014	NJAS-Wageningen Journal of Life Science
Rehabilitation of individuals on long-term sick leave due to sustained stress-related symptoms: A comparative follow-up study	Willert et al.	2014	Scandinavian Journal of Public Health
Changes in experienced value of everyday occupations after nature-based vocational rehabilitation	Pálsdóttir et al.	2014	Scandinavian Journal of Public Health
Agriculture- Who cares? An Investigating of care farming in the UK	Leck et al.	2014	Journal of Rural Studies
Nature-assisted rehabilitation for reactions to severe stress and/or depression in a rehabilitation for reactions to severe stress and/or depression in a rehabilitation garden long-term follow-up including comparisons with a matched population-based reference cohort	Wāhrborg et al.	2014	Journal of Rehabilitation Medicine
The journey of recovery an empowerment embraced by nature. Client´s perspective on nature-based rehabilitation in relation to the role of the natural environment	Pálsdóttir et al.	2014	International Journal of Environmental Research and Public Health
Narratives of natural recovery: Youth experience of social inclusion though garden care	Kogstad et al.	2014	International Journal of Environmental Research and Public Health
Outsourcing mental health care services? The practice and potential of community-based Farms in psychiatric rehabilitation	Iancu et al.	2014	Community Ment Health J
Understanding the impacts of care farms on health and well-being of disadvantage populations: a protocol of the Evaluating Community Orders (ECO) pilot study	Elsey et al.	2014	BMJ Open
Decentralisation of long-term care in Netherlands: the case of day care at green care farms for people with dementia	Nowak et al.	2015	Ageing and Society
Can rehabilitation in boreal forests help recovery from exhausting disorder? The randomised clinical trial forest	Sonntag-Öström et al.	2015a	Scandinavian Journal of Forest Research
"Nature´s effect on my mind" Patient´s qualitative experiences of a forest-based	Sonntag-Öström et al.	2015b	Urban Forestry and Urban Greening

Title	Authors	Publication year	Journal
rehabilitation programme			
Diversification and re-feminisation of Norwegian Farm Properties	Heggem	2015	Sociologia Ruralis
Green care from the provider's perspective: An insecure position facing different social worlds	Lund et al.	2015	SAGE Open
New practices of farm-based community-oriented social care services in The Netherlands	Hassink et al.	2015	Journal of Social Service Research
Guidelines for the design of a healing garden for the rehabilitation of psychiatric patients	Erbino et al.	2015	Journal of Agricultural Engineering
Farming families as foster families: The findings of an exploratory study on care farming in Switzerland	Bombach et al.	2015	International Journal of Child, Youth and Family Studies
Using nature-based rehabilitation to restart a stalled process of rehabilitation in individuals with stress- related mental illness	Shalin et al.	2015	International Journal of Environmental Research and Public Health
The significance of experiences of nature for people with Parkinson's Disease, with special focus on freezing of gait—The necessity for a biophilic Environment. A multi-method single subject study	Ottosson et al.	2015	International Journal of Environmental Research and Public Health
Sowing the seeds or failing to blossom? A feasibility study of a simple ecotherapy-based intervention in women affected by breast cancer	Phelps et al.	2015	Ecancermedicalscience
Growing well-beings: The Positive experience of care farms	Leck et al.	2015	British Journal of Health Psychology
Living at the farm, innovating nursing home care for people with dementia- study protocol of an observational longitudinal study	De Boer et al.	2015	BMC Geriatrics
Potential use of *Mucor circinelloides* for the biological control of certain helminths affecting livestock reared in a care farm	Cortiñas et al.	2015	Biocontrol Science and Technology
Farm animal-assisted intervention for people with clinical depression: A randomized controlled trial	Pedersen et al.	2015	Anthrozoös
Motivations, experiences and challenges of being a care farmer- results of a survey of Norwegian care farmers	Ihlebaeck et al.	2016	Work-A Journal of Prevention Assessment and Rehabilitation
Autonomy support and need satisfaction in prevocational programs on care farms: the self-determination theory perspective	Ellingsen-Dalskau et al.	2016	Work-A Journal of Prevention Assessment and Rehabilitation
Content and key components of vocational rehabilitation on care farms for unemployed people with mental health problems: A case study report	Pedersen et al.	2016	Work-A Journal of Prevention Assessment and Rehabilitation
Social farming in the promotion of social-ecological sustainability in rural and periurban areas	García-Llorente et al.	2016	Sustainability
Gardening is beneficial for adult mental health: Scottish Health Survey, 2012-2013	Shiue	2016	Scandinavian Journal of Occupational Therapy
What does care farming provide for clients? The views of care farm staff	Hemingway et al.	2016	NJAS- Wageningen Journal of Life Science

Title	Authors	Publication year	Journal
Design of evidence-based gardens and garden therapy for neurodisability in Scandinavia: data from 14 sites	Spring	2016	Neurodegenerative Disease Management
Entrepreneurship in agriculture and healthcare: Different entry strategies of care farmers	Hassink et al.	2016a	Journal of Rural Studies
Identity formation and strategy development in overlapping institutional fields. Different entry & alignment strategies of regional organizations of care farms into the healthcare domain	Hassink et al.	2016b	Journal of Organizational Change Management
The role of soundscape in nature-based rehabilitation: A patient perspective	Cerwen et al.	2016	International Journal of Environmental Research and Public Health
Measuring the effects of transdisciplinary research: the case of a social farming project	Di Iacovo et al.	2016	Futures
Understanding how prevocational training on care farms can lead to functioning, motivation and well-being	Ellingsen-Dalskau et al.	2016	Disability and Rehabilitation
Green fingers and clear minds: prescribing "care farming" for mental illness	Elsey et al.	2016	British Journal of General Practice
The influence of therapeutic horticulture on social integration	Howart et al.	2016	Journal of Public Mental Health
From green space to green prescriptions: challenges and opportunities for research and practice	Van der Berg	2017	Frontiers in Psychology
Care farms as a space of wellbeing for people with a learning disability in the United Kingdom	Rotheram et al.	2017	Health and Place
Social farming in Catalonia: Rural local development, employment opportunities and empowerment for people at risk of social exclusion	Guirado et al.	2017	Journal of Rural Studies
Exploring the role of farm animals in providing care at care farms	Hassink et al.	2017	Animals
Green care farms as innovative nursing homes, promoting activities and social interaction for people with dementia	de Boer et al.	2017a	Journal of the American Medical Directors Association
Quality of care and quality of life of people with dementia living at green care farms: a cross-sectional study	de Boer et al.	2017b	BMC Geriatric
Smelling therapeutic landscapes: Embodied encounters within spaces of care farming	Gorman and Cacciatore	2017	Health and Place
The location matters: Determinants for "deepening" and "broadening" diversification strategies in Ruhr metropolis' urban farming	Pölling and Mergenthaler	2017	Sustainability
Collaboration, knowledge and innovation toward a welfare society: the case of the Board of Social Farming in Valdera (Tuscany), Italy	Di Iacovo et al.	2017	Journal of Agricultural Education and Extension
Business models in urban farming: a comparative analysis of case studies from Spain Italy and Germany	Pölling et al.	2017	Moravian Geographical Reports
The development of social farming in Italy: A	Dell´Olio et al.	2017	Journal of Rural Studies

Title	Authors	Publication year	Journal
qualitative inquiry across four regions			
The social dimensions of therapeutic horticulture	Harris	2017	Health and the Social Care in the Community
Longer nature-based rehabilitation may contribute to a faster return to work in patients with reactions to severe stress and/or depression	Grahn et al.	2017	International Journal of Environmental Research and Public Health
A diagnostic post-occupancy evaluation of the Nacadia Therapy Garden	Sidenius et al.	2017	International Journal of Environmental Research and Public Health
`The nourishing soil of the soul': The role of horticultural therapy in promoting well-being in community-dwelling people with dementia	Noone et al.	2017	Dementia
Stress recovery in forest or handicraft environments – An intervention study	Dolling et al.	2017	Urban Forestry and Urban Greening
Rural–urban business partnerships-Towards a new trans-territorial logic	Hjalager	2017	Local Economy
Therapeutic landscapes and non-human animals: the roles and contested positions of animals within care farming assemblages	Gorman	2017	Social and Cultural Geography
